# Catatonia Associated with Hyponatremia: Case Report and Brief Review of the Literature

**DOI:** 10.2174/1745017902117010026

**Published:** 2021-05-24

**Authors:** Vaios Peritogiannis, Dimitrios V. Rizos

**Affiliations:** 1 Mobile Mental Health Unit of the prefectures of Ioannina and Thesprotia, Society for the Promotion of Mental Health in Epirus, Ioannina, Greece; 2 Intensive Care Unit, “Hatzikosta” General Hospital, Ioannina, Greece

**Keywords:** Benzodiazepines, Catatonia, Delirium, Hyponatremia, Psychogenic polydipsia, Schizophrenia-spectrum disorders

## Abstract

**Background:**

Catatonia is a syndrome of altered motor behavior that is mostly associated with general medical, neurologic, mood and schizophrenia-spectrum disorders. The association of newly onset catatonic symptoms with hyponatremia has been rarely reported in the literature.

**Case Presentation:**

We present a rare case of a young female patient with schizophrenia, who presented with catatonic symptoms in the context of hyponatremia due to water intoxication. The symptoms were eliminated with the correction of hyponatremia. There are only a few reports of hyponatremia-associated catatonia in psychiatric and non-psychiatric patients. Sometimes, catatonic symptoms may co-occur with newly onset psychotic symptoms and confusion, suggesting delirium. In several cases, the catatonic symptoms responded to specific treatment with benzodiazepines or electroconvulsive therapy.

**Conclusion:**

Hyponatremia may induce catatonic symptoms in patients, regardless of underlying mental illness, but this phenomenon is even more relevant in patients with a psychotic or mood disorder, which may itself cause catatonic symptoms. It is important for clinicians not to attribute newly-onset catatonic symptoms to the underlying psychotic or mood disorder without measuring sodium serum levels. The measurement of sodium serum levels may guide treating psychiatrists to refer the patient for further investigation and appropriate treatment.

## 1 INTRODUCTION

Catatonia is a syndrome of altered motor behavior characterized by mutism, posturing, negativism, staring, rigidity, echophenomena (*i.e.*, echolalia or echopraxia), stupor, and lack of response to pain [1]. Catatonia may accompany several general medical and neurologic disorders, and it is more frequently associated with mood disorders and neurotoxic syndromes than with schizophrenia [2]. Conceivably, catatonic syndrome is encountered in various clinical settings, besides psychiatric wards, such as the consultation-liaison psychiatry [3], the emergency department [4], and the intensive care unit [5, 6].

Hyponatremia (serum sodium concentration <135mEq/L) is the most common electrolyte abnormality encountered in clinical settings [7]. It is also the most common electrolyte disorder detected in patients with psychotic disorders, with estimated prevalence rates ranging from 7%-10% among inpatients [8]. Patients with acute hyponatremia may display severe symptoms, such as confusion, seizures, and coma. In non-acute and mild cases of hyponatremia, symptoms are less severe and may be vague, such as fatigue, lethargy, headache, and irritability, which may be difficult to distinguish from symptoms of psychosis itself [9].

The association of hyponatremia with the development of catatonic symptoms may have important clinical implications, yet it has been rarely reported in the literature. Here we report a case of catatonia which had been developed in the context of hyponatremia in a female patient with schizophrenia. Subse- quently, a brief review of the published cases of hyponatremia-induced catatonia in psychiatric and non-psychiatric patients is presented.

## 2 CASE PRESENTATION

A 41-year-old female patient with an 18-year history of schizophrenia was referred to the emergency department of the local general hospital. She was presented with mutism, staring gaze, rigidity, negativism, and stupor. The symptoms developed over a period of 3 days. The patient had no history of general medical illness or alcohol/substance abuse. She had been treated in a community mental health service [10] for several years and was clinically stable during the last semester under treatment with haloperidol decanoate 150mg monthly. According to the information given by her family, she was drinking excessive amounts of water for several days prior to the development of the catatonic symptoms. The patient was admitted to the internal medicine ward for further evaluation and treatment. At admission, she was afebrile and awake, with no alteration of consciousness or other signs of confusion. A full clinical and laboratory investigation was employed. The computed tomography of the brain was normal, as well as the electroencephalogram. The blood sample analysis revealed moderate hyponatremia, with sodium levels 124mEq/L. The urine analysis did not reveal any significant findings. The patient received treatment with water restriction and isotonic saline and was discharged in 2 days, in good clinical condition, with sodium serum levels 138mEq/L. The physicians’ instructions at discharge were water restriction, laboratory re-examination, and psychiatric evaluation. She was examined by the treating psychiatrist the next day of discharge and did not present any signs of catatonia. Her clinical condition was stable over the following weeks, and sodium serum levels were 138mEq/L 4 weeks after discharge.

### 2.1 Review of the Literature

A search in the PubMed database revealed 10 previous reports of the hyponatremia-associated catatonic syndrome, involving 11 patients which are summarized in Table **1**. Seven cases involved patients with a mental disorder. The first report was from India and involved a male patient with bipolar disorder. The patient presented catatonic symptoms (mutism, negativism, rigidity) in the context of severe hyponatremia of unknown cause. All symptoms abated with the restoration of sodium serum levels [11]. Maxwell *et al.* [12] reported two cases of young females who presented with catatonic stupor after the consumption of 3,4-methylenedioxymethamphetamine (ecstasy) and the development of hyponatremia. Both recovered completely within several hours with conservative management. A subsequent report involved a 45-year-old male patient with schizophrenia, who developed catatonic symp- toms, such as grimacing, posturing, stereotypy, and negativism, which were associated with hyponatremia. The patient had symptoms of confusion as well. The administration of lorazepam was employed for the resolution of the catatonic symptoms [13]. In another report of hyponatremia-associated catatonia, a young male patient with schizophrenia was presented with stupor, staring gaze, akinesia, negativism, mutism, and muscular rigidity. The patient had suffered a brain injury that had been accounted for the development of hyponatremia, although he had a normal brain image and no neurological sequelae. All symptoms resolved with the correction of hypo- natremia [14]. There is also a reported case of hyponatremia-induced persistent and recurrent catatonic syndrome in a non-psychiatric patient due to adrenal insufficiency. The symptoms did not respond to serum sodium levels restoration or lorazepam, and a course of electroconvulsive therapy (ECT) was required for symptom resolution [15]. On other occasions, a middle-aged female patient developed catatonic symptoms (immobility, withdrawal, rigidity, negativism, mutism, and posturing) and delirium in the context of hyponatremia induced by venlafaxine. Treatment with lorazepam improved the symptoms, which were completely resolved with the correction of hyponatremia [16]. Novac *et al.* [17] reported the case of a young female patient with depression and papillary thyroid cancer, who was presented with confusion, psychosis, and catatonic symptoms due to hyponatremia in the context of iodine-131 therapy for metastatic cancer. Treatment with clonazepam and the restoration of sodium serum levels improved the catatonic symptoms. In another report, a female patient with schizoaffective disorder and medical multi-morbidity was presented with stupor, mutism, posturing, stereotypy, and staring due to hyponatremia secondary to psychogenic polydipsia. The catatonic symptoms resolved with lorazepam administration [18]. A unique case of a 69-year-old male patient who developed two discrete episodes of catatonia associated with hyponatremia was reported by McGuire *et al.* [19] Symptoms of confusion and psychosis were recorded as well. The two episodes had taken place over a 3-year interval. During the first presentation of the patient, treatment with diazepam resolved the catatonic symptoms, whereas on the second occasion, the correction of sodium serum levels sufficed for symptom resolution. Finally, there is a recent case of the development of catatonic symptoms in an elderly male patient in the context of hyponatremia induced by imiquimod treatment that the patient had received for actinic keratosis. The catatonic symptoms improved with lorazepam [20].

## 3 DISCUSSION

This is a rare case of hyponatremia-associated catatonia in a patient with a schizophrenia-spectrum disorder. In this case, the patient developed catatonic symptoms rapidly, probably due to water intoxication, as a result of the so-called psychogenic polydipsia. Psychogenic polydipsia is a well-known phenomenon in patients with schizophrenia-spectrum disorders, with a reported incidence of 11 to 20% in chronic patients [21]. The pathophysiology of psychogenic polydipsia is complex and poorly understood, although it has been observed to worsen during acute psychotic episodes. It has been hypothesized that during acute psychosis, the activation of the hypothalamic-pituitary-adrenal axis and the hormone arginine vasopressin secretion influences polydipsic behavior [22]. However, in this case, the patient had been clinically stable for several months. In this case, the rapid recovery of the patient may be due to the relatively young age (41 years) and the absence of any significant medical comorbidities. Moreover, the resolution of hyponatremia was sufficient to resolve the catatonic symptomatology rapidly, without further interventions. However, in several cases, the application of specific interventions, such as lorazepam or other benzo- diazepine administration [13, 16-20], or ECT[15], was required for the resolution of catatonic symptoms.

The limited evidence that was presented here suggests that hyponatremia may induce catatonic symptoms in patients, regardless of underlying mental illness. However, this phenomenon is even more relevant in patients with a psychotic or mood disorder, which may itself cause catatonic symptoms. It is important not to attribute newly-onset catatonic symptoms to the underlying psychotic or mood disorder without measuring sodium serum levels. The measurement of sodium serum levels with a blood test is simple and may guide treating psychiatrists to refer the patient for further investigation and appropriate treatment. Importantly, rapid and inappropriate correction of hyponatremia may induce catatonic syndrome as well. Central pontine myelinolysis is an acute demyelinating neurological disorder affecting the central pons primarily and is frequently associated with rapid correction of hyponatremia. There is some evidence that central pontine myelinolysis may occasionally present with neuropsychiatric manifestations, including catatonia [23, 24]

In cases of multi-morbidity, it may not be clear whether hyponatremia is the single cause of catatonic syndrome. Presumably, multiple causal factors may act synergistically to cause catatonia. Clinicians should be aware of the general medical conditions that may induce catatonic symptoms. The co-existence of a severe mental health disorder, such as schizophrenia, or psychotic depression, may further predispose medical patients to develop the catatonic syndrome. It is worthy of mentioning that, apart from causes of hyponatremia related to the psychotic disorder itself, such as psychogenic polydipsia, treatment-related factors should also be considered as causes of hyponatremia and possible catatonia in psychotic or mood disorders. It has been suggested that both antipsychotic medications and antidepressants have the potential to cause hyponatremia *via* the induction of the syndrome of inappropriate antidiuretic hormone secretion [25]. Indeed, in one of the reported cases, treatment with venlafaxine had been accounted for the hyponatremia-associated catatonia [16]. Moreover, some antiepileptic drugs that are employed as antimanic agents and mood stabilizers in the treatment of bipolar disorder may also cause hyponatremia. In a large population-based case-control study in Sweden, it was found that there was a strong association between newly initiated treatment with carbamazepine and hospitalization due to hyponatremia. The association of valproate treatment with hyponatremia was moderate, whereas lamotrigine had the lowest risk of both during initiation and ongoing treatment [26]. Importantly, lithium treatment has been inversely associated with hospitalization due to hyponatremia, and this effect is probably mediated by lithium-induced nephrogenic diabetes insipidus [27].

The underlying mechanisms of catatonia are still poorly understood, although it has been conceptualized as a disorder of cerebral motor network dysfunction. Indeed, neuroimaging studies suggest that catatonia is associated with alterations of cerebral motor circuits in patients with schizophrenia [28]. It has been suggested that alterations in GABAergic and glutamatergic neural activity may contribute to some but not all phenotypes of catatonia [29], whereas other research has highlighted the role of the activation of the innate immune system in the development of catatonic symptoms [30]. Similarly, mechanisms of hyponatremia-induced catatonia remain elusive. It is uncertain whether the proposed underlying mechanisms of catatonia in patients with schizophrenia are relevant in cases of hyponatremia-induced catatonia, or other cases of catatonia associated with a general medical condition.

It should be noted that in several cases of hyponatremia-associated catatonia in non-psychotic patients [17, 19], the catatonic symptoms accompanied newly onset psychotic symptoms. These symptoms seemed to have been developed in the context of a confusional state. The co-occurrence of the syndromes of catatonia and delirium has received research attention in recent years in a variety of clinical settings [31, 32]. In patients with an established psychotic-spectrum disorder, psychotic symptoms may be attributed to decompensation of the primary disorder. It is important for clinicians to detect any signs of confusion, such as altered consciousness and disorientation, which would guide diagnostic workup toward an organic cause.

## CONCLUSION

Hyponatremia may be a rare but treatable cause of catatonia, and it should be suspected in cases of newly onset catatonic symptoms in psychiatric and non-psychiatric patients. Clinicians should be aware of the hyponatremia/catatonia relationship and monitor serum electrolytes appropriately in catatonic patients. It is particularly important to consider hyponatremia as a potential cause of catatonia in patients with an established psychotic disorder. The correction of hyponatremia may not always suffice for the elimination of the catatonic syndrome, and treatment with benzodiazepines or ECT may be warranted.

## Figures and Tables

**Table 1:** Cases of hyponatremia-induced catatonic syndrome

Case	Patient’s gender and age (in years)	Patients’ mental health history	Serum sodium (mEq/L)	Cause of hyponatremia	Comments
Nizamie *et al.* [11]	M, 45	Bipolar disorder	116	Unclear	The catatonic symptoms were resolved with the correction of hyponatremia
Maxwell *et al.* [12]	F, 17 F, 17	None None	130 118	Unclear. Both had taken 3,4-methylenedioxy- methamphetamine (ecstasy)	Both recovered within 12 and 18 hours, respectively
Lee & Schwartz [13]	M, 45	Schizophrenia	113	Water intoxication	Delirium was present. Treatment with lorazepam improved the catatonic symptoms before sodium levels restoration
Yeh *et al.* [14]	M, 36	Schizophrenia	120	Cerebral salt wasting syndrome, associated with TBI	Catatonic symptoms resolved when the hyponatremia improved
Grover *et al.* [15]	F, 48	None	109	Adrenal insufficiency	Symptoms of catatonia did not respond to correction of hyponatremia or lorazepam. Resolution with ECT
Grover *et al.* [16]	F, 59	Recurrent depression	127	Venlafaxine	Delirium was also present. Symptoms of catatonia responded to lorazepam and resolved with the correction of hyponatremia
Novac *et al.* [17]	F, 30	Depression	110	Radio-contrast iodine therapy	Confusion was evident. The catatonic symptoms resolved with correction of sodium serum levels plus clonazepam
Krueger *et al.* [18]	F, 43	Schizoaffective disorder	127	Psychogenic polydipsia	Catatonic symptoms did not resolve with the correction of hyponatremia but with lorazepam treatment
McGuire *et al.* [19]	M, 69	Depression	125 and 116	Unclear	This case describes two episodes of hyponatremia-induced catatonia in the same patient. Confusion was recorded as well. Symptom resolution was associated with correction of sodium imbalance, plus diazepam treatment
Little & Cheng [20]	M, 81	None	125	Imiquimod treatment	Catatonic symptoms improved with lorazepam
Our case	F, 41	Schizophrenia	124	Water intoxication	Complete resolution of symptoms with sodium serum levels correction

F: female, M: male, ECT: electroconvulsive treatment, TBI: traumatic brain injury
